# Enhancing cancer clonality analysis with integrative genomics

**DOI:** 10.1186/1471-2105-16-S13-S7

**Published:** 2015-09-25

**Authors:** Erich A Peterson, Michael A Bauer, Shweta S Chavan, Cody Ashby, Niels Weinhold, Christoph J Heuck, Gareth J Morgan, Donald J Johann

**Affiliations:** 1 Myeloma Institute, University of Arkansas for Medical Sciences, Little Rock, AR, USA

## Abstract

**Introduction:**

It is understood that cancer is a clonal disease initiated by a single cell, and that metastasis, which is the spread of cancer from the primary site, is also initiated by a single cell. The seemingly natural capability of cancer to adapt dynamically in a Darwinian manner is a primary reason for therapeutic failures. Survival advantages may be induced by cancer therapies and also occur as a result of inherent cell and microenvironmental factors. The selected "more fit" clones outmatch their competition and then become dominant in the tumor via propagation of progeny. This clonal expansion leads to relapse, therapeutic resistance and eventually death. The goal of this study is to develop and demonstrate a more detailed clonality approach by utilizing integrative genomics.

**Methods:**

Patient tumor samples were profiled by Whole Exome Sequencing (WES) and RNA-seq on an Illumina HiSeq 2500 and methylation profiling was performed on the Illumina Infinium 450K array. STAR and the Haplotype Caller were used for RNA-seq processing. Custom approaches were used for the integration of the multi-omic datasets.

**Results:**

Reported are major enhancements to CloneViz, which now provides capabilities enabling a formal tumor multi-dimensional clonality analysis by integrating: i) DNA mutations, ii) RNA expressed mutations, and iii) DNA methylation data. RNA and DNA methylation integration were not previously possible, by CloneViz (previous version) or any other clonality method to date. This new approach, named iCloneViz (integrated CloneViz) employs visualization and quantitative methods, revealing an integrative genomic mutational dissection and traceability (DNA, RNA, epigenetics) thru the different layers of molecular structures.

**Conclusion:**

The iCloneViz approach can be used for analysis of clonal evolution and mutational dynamics of multi-omic data sets. Revealing tumor clonal complexity in an integrative and quantitative manner facilitates improved mutational characterization, understanding, and therapeutic assignments.

## Background

It is recognised that cancer is a clonal disease instigated by a single cell and that metastasis is also commenced thru a single cell [1-3]. Tumors are composed of a variety of clones or subpopulations of cancer cells that may differ, for instance in their expression of cell surface markers, sensitivity to therapeutic agents, karyotype, proliferation rate. A cancer clone or subclone is a cell or group of cells that have formed from an original cell as a result of a new mutation [4]. Many cancers including multiple myeloma (MM) are difficult to treat due to their dynamic adaptability resulting from clonal evolution [5].

Evolution is an important scientific concept because it works. It provides a framework to explain changes in biological systems. Cancer is the result of an evolutionary process, but it is destructive, since it involves the loss of mechanisms that are implemented to protect against uncontrolled and undifferentiated growth. Ultimately, natural selection has a harsh reality that worried Darwin, namely, all that seems to matter is reproductive success [6].

MM is a cancer of the bone marrow characterized by a malignant transformation and proliferation of plasma cells [7]. Definitive therapies include combination chemotherapy, autologous transplant regimens [8], and two new classes of agents called immunomodulatory drugs (IMiDs) and proteasome inhibitors [9-11]. A significant improvement in patient survival has occurred over the last ~15 years [12]. However, there is still significant variation in outcome and an explanation is tumor heterogeneity with associated complex genomic landscapes [5,13,14].

There exist an array of computational methods/tools that allow one to characterize various aspects of the clonal architecture of a tumor(s). Each method employs different computational and visualization techniques, and are very briefly described. SciClone [15] allows for the characterization of the clonal structure of a tumor using multiple samples, in an attempt to shed light on "cryptic subclones", which can appear when only one sample is analyzed. Several visualizations are available within SciClone, and clustering on the variant allele frequencies of somatic mutations (using a variational Bayesian mixture model on copy number neutral regions) attempts to infer the number and composition of subclones. PyClone [16] makes use of hierarchical Bayesian clustering models to estimate the number and cellular prevalence of subclones from the variant allele frequencies of somatic mutations. The method also takes into account copy number variation and possible normal cell contamination in the model. TrAp [17], in addition to inferring the subclones and their abundances within a single "aggregate sample" (via "aberrant frequencies" aka variant allele frequencies), constructs a phylogenetic tree--describing the evolution of clones within the tumor. The problem is modeled, and solutions based mathematically, on the deconvolution of a single aggregate signal.

Clomial [18] is another method used to infer subclonal structure using a binomial expectation maximization based approach (via somatic variant allele frequencies), and was specifically designed to deal with multiple samples from a single tumor sample. Rec-BTP [19] casts the problem of uncovering the clonal structure of a single sample, using the variant allele frequencies of somatic mutations, as a combinatorial one. A recursive algorithm using a binary tree partition is developed which approximates the originally formulated NP-complete problem. THetA [20] uses copy number aberrations to discover most likely clonal subpopulations. In particular, the problem of subclonal characterization is solved using a maximum likelihood mixture decomposition method, in order to find the genotypes "whose mixture best explains the observed sequencing data." PhyloWGS [21] is unique, in that it makes use of both somatic mutational and copy number variation data in its subclonal analysis. It additionally infers a phylogenetic tree (using non-parametric mathematical approaches) explaining the evolutionary history of a tumor's clonal composition, as well as, each clone's relative abundance. The method is also able to take multiple sample inputs to aid subclonal reconstruction.

All of the aforementioned referenced methods only make use of a single modality in their characterizations of clonal architecture, namely, DNA-based mutational data, culled from whole genome or whole exome sequencing (WES) experiments. Some methods also attempt to account for the effect of copy number variation on clonal architecture. This is contrasted to the multiple modality datasets employed in the characterization of clonal structure used within iCloneViz. iCloneViz is the only known computational method for inferring clonal architecture, which integrates multiple modality datasets to derive deeper biological meaning. For example, RNA variant calling is performed (a relatively new method of variant calling) to detect whether or not a mutation found within the DNA (via a WES or WGS experiment) is detectable within a RNA transcript. Further, if a mutation is found to be present at the RNA level, the expression values associated with the transcript(s) containing the mutation can be quantified and visualized. Finally, DNA methylation data is integrated into the analysis, which could lead to hypotheses regarding methylation suppressing the expression of a tumor suppressor gene (TSG). iCloneViz tracks TSGs both by mutations and epigenetic/methylation events.

In the presented analysis, integrated clonal dynamics from a single patient diagnosed with MM is explored. Tumor material is investigated at initial diagnosis (i.e., *Presentation*) and then later when the cancer recurred (i.e., *Relapse*). A novel bioinformatic approach named CloneViz [22] has been enhanced to allow for the ***rapid integration, quantitation, visualization***, and ***investigation ***of the mutational dynamics from **i**) WES, **ii**) RNA-seq, and **iii**) DNA methylation.

The methodology is independent of any specific cancer type and MM is used as a demonstrative example due to its intrinsic heterogeneity. The novelty of the approach concerns the rapid integration and dissection of large and complex multi-omic datasets. Discovered in this study is first, the existence of an amplified and mutated *MYC *oncogene in both *Presentation *and *Relapse*. Second, the occurrence of hotspot mutations in *MTOR *with rationale to treat, and in *KRAS*, with evidence not to treat. Third, is progressive evidence of tumor suppressor gene silencing. Overall, the molecular profiling and advanced bioinformatics (iCloneViz) provide for a more precise understanding of tumorigenesis with potential for an improved outcome via precision medicine-based approaches.

## Methods

### Sample and library preparation

All samples were obtained from a single patient with MM at the Myeloma Institute, University of Arkansas for Medical Sciences (UAMS), Little Rock, AR. Sample collection protocols were approved by the UAMS Institutional Review Board (IRB). Collected samples include bone marrow aspirate with biopsy and peripheral blood. Tumor plasma cells were obtained from the bone marrow aspirate after enrichment by anti-CD 138 immuno-magnetic bead selection at a central laboratory, in a manner previously described [23]. Selected tumor material had a purity greater than 96% for the presentation sample and 99% for relapse. CD-138 is a marker for malignant plasma cells in a patient with clinical MM. The patient's first (*Presentation*) and second (*Relapse*) tumor samples were obtained in 2010 and 2013 respectively. Normal (germline) material was obtained from the buffy coat of peripheral blood in 2010, after density gradient centrifugation. To ensure the absence of plasma cells buffy coat material was also examined by flow cytometry.

Tumor and normal whole exome libraries were constructed from 50 ng of DNA material after shearing, end repair, phosphorylation, and ligation to bar coded sequencing adapters. DNA material was further fragmented using the S220 focused-ultrasonicator (Covaris), for a target base pair (bp) length of 300. DNA was size selected for lengths between ~ 250 - 330 bp. DNA regions were captured using the Agilent SureSelect^QXT ^Human All Exon v5 Plus hybrid capture kit. Samples were then multiplexed and subjected to sequencing (101 bp paired-end reads) on an Illumina HiSeq 2500. Whole exome libraries were sequenced to an average depth of 100x.

RNA-seq tumor libraries were constructed using 200 ng of total RNA material, using the Illumina TruSeq mRNA v2 kit according to the manufacturer's instructions. Poly-A selection for mRNA was first performed using streptavidin-coated magnetic beads, which was followed by thermal mRNA fragmentation. mRNA was then subjected to cDNA synthesis using reverse transcriptase. Resulting cDNA was converted to double stranded cDNA, followed by end repair, and then ligated to paired-end adapters. Size selection was performed using AMPure XP beads (Beckman Coulter), for sequences ~ 300 - 350 bp in length. The library was further enriched using 15 cycles of PCR and purified again with AMPure XP beads. The concentration of material run was 8 pM. The libraries were then multiplexed and subjected to sequencing (101 bp paired-end reads) on an Illumina HiSeq 2500. RNA-seq libraries were sequenced to a total of 100M reads.

Genome-wide DNA methylation was assessed in bisulfite-converted genomic DNA using the Illumina Infinium HumanMethylation450 (HM450K) BeadChip array, which contains 485,577 probes covering 99% of RefSeq genes, 96% of CpG islands (CGI) and coverage across promoters, 5' and 3'-UTRs, first exons and gene bodies. Genomic DNA (500 ng) was bisulfite treated and purified using the EZ DNA Methylation-Gold kit (Zymo Research, Irvine, CA) according to the manufacturer's protocol. The resultant bisulfite-converted DNA was processed, hybridized to Illumina HumanMethylation450 BeadChips, fluorescently stained and scanned according to the Infinium HD Assay Methylation Protocol User's Guide provided by Illumina. Processed BeadChips were scanned on an Illumina iScan and methylation values were determined for all probes using the GenomeStudio Methylation module (Illumina).

### Whole exome sequencing processing

FASTQ file generation and demultiplexing from BCL files was performed using CASAVA v1.8.2 [24]. Quality control and assessment was performed on FASTQ files using FastQC v0.11.2 [25]. Unidentified bases at read ends (i.e., those recorded as 'N') were removed using a custom utility--creating final read lengths between 93 to 101 bps. Each sample's FASTQ paired-end files were aligned to the Ensembl reference genome (build GRCh37.75) using a hybrid approach that employed BWA v0.7.12 [26] and then STAMPY v1.0.22 [27]. Quality control and assessment of the aligned sequence alignment/map (binary alignment/map) SAM/BAM files were assessed with QualiMap v2.0.2 [28]. SAM/BAM post-processing steps were performed to mark duplicates, add read group information, sort, and reorder aligned reads (Picard Tools v1.119 [29]). GATK v3.3-0 [30] was used to perform local realignment and base quality recalibration. Copy number data was computational inferred using ExomeCNV v1.4 [31]. Single nucleotide variants (SNVs) and small insertions and deletions (InDels) were called using Strelka v1.0.14 [32] (tumor and normal pairs), resulting in variant call format (VCF) files. SnpEff v4.0e [33] was used to annotate each variant with its predicted functional effects.

### RNA sequencing processing

#### Transcriptome reconstruction

RNA-seq samples were first demultiplexed and FASTQ files were created from BCL files using CASAVA v1.8.2. Quality control and assessment was performed on FASTQ files using FastQC v0.11.2. Trimmomatic v0.32 [34] (utilizing a sliding window approach) was used to trim low quality reads and remove possible adapter sequences. Alignment of reads and transcriptome reconstruction was performed using the Tuxedo suite of tools. TopHat v2.0.12 [35] was used to align each sample's paired-end reads to the Ensembl reference genome build GRCh37.75. Quality control and assessment of resulting BAM files was performed using SAMtools v0.1.8 [36]. BAM files were then used to reconstruct the transcriptome and quantify each isoform's fragments per kilobase of transcript per million mapped reads (FPKM) using Cufflinks v2.2.1 [37], and Cufflinks was run in a mode which allows for the discovery of novel isoforms. HTSeq v0.6.1 [38] was used to quantify raw (non-normalized) gene-based read counts.

#### RNA-seq based variant calling

After demultiplexing and creating FASTQ files using the previous subsection's description, RNA variants were called using the Broad Institute's GATK Best Practices for RNA-seq variant calling [39]. These steps include the following: STAR v2.3.0e [40] was used to align reads to the Ensembl reference genome (build GRCh37.75), using the recommended "2-pass" approach. Duplicates were marked and the aligned reads sorted with Picard Tools. Next, the tool SplitNCigarReads (GATK component) was used to split reads into exon segments, clip reads which overhang intronic regions, and assign a default MAPQ score of 60 to all reads. Variants were called using the HaplotypeCaller tool (GATK component).

### Variant quantification and classification

The variant allele frequency (VAF) was determined by dividing the total reads for the variant (TRV) by the sum of the total reads for the variant (TRV) plus total reads for the reference (TRR). Copy number data was derived using ExomeCNV v1.4. The selection and retention of variants were based on the following filtering parameters: i) VAF ≥ 4%, and ii) 20 ≤ DP ≤ 1000. A manual evaluation of the read alignments using the Integrative Genomics Viewer (IGV) v2.3.32 was also performed [41]. At times a second selection of variants utilized an intersection against a key gene (KG) list. The KG group was constructed from the following public sources: **i) **known drivers and cancer predisposition genes cited in Vogelstein, et. al. [42], **ii) **Foundation One Heme™ Genes (http://foundationone.com/genelist2.php) and, **iii**) the MD Anderson listing of human DNA repair genes [43].

### Quantifying clonal diversity

Diversity measures from ecology were adapted to quantify clonal diversity in MM serial samples [44]. Each sample is not a single organism/species, but rather consists of thousands of cells from a purified bone marrow aspirate. The abundance of a molecular species (variant/mutation) is the product of VAF * DP * CN. The number of clones in a neoplasm is a simple measure of diversity. Diversity measures typically incorporate both the number and abundance of clones [44]. The Shannon diversity index (*SDI*) [45] is

(1) $$SDI=-\sum_{i}^{N}p\left(i\right)ln\left(p\left(i\right)\right)$$

where *p(i) *is the frequency of clone *i *in the neoplasm.

The SDI computes a single quantitative value based on the number of different mutations in the cancer sample and how evenly distributed each mutation is among the entire group. The SDI value will increase when the number of distinct mutations increases and also when the evenness among the mutations increases [44]. There are other diversity measures (e.g., Simpson, Berger-Parker) but, the Shannon diversity index is preferable because it is not dominated by the most frequent clone, and it has been utilized in previous studies of cancer [46,47].

### Software engineering and integrative analysis

This section and its constituent sub-sections documents and describes all software components, data structures, and algorithms used to construct the extended and integrated CloneViz (iCloneViz), especially the data calculations, visualizations and the rationale for their use.

#### General technology and frameworks

The C# programming language, targeting the .NET Framework v4.5 [48] (utilizing the integrated development environment Visual Studio 2013 [49]), was used to construct iCloneViz. This programming platform was used to facilitate the rapid development of an interactive Windows-based application. To minimize the amount of custom data access layer code needed to perform the data-intensive functions of iCloneViz, the object-relational mapping software Entity Framework v6.1.1 [50] (using the database-first model) was utilized along with the molecular profiling modality database (MPMDB), which is further described later in this study. The built-in .NET Framework charting package (i.e., namespace System.Windows.Forms.DataVisualization.Charting), was used to provide visualization primitives. This package was used in favor of others, because of its relative ease-of-use and built-in functions, in particular, its ability to perform smart label positioning. The relational database management system SQL Server 2012 Developer Edition [51] was used to store all patient data and meta-data. The database system was chosen for its robustness, ability to scale, and its low cost for academic and research use. (The database system is described in further detail in the "Data storage, retrieval and annotation" section below).

#### Mathematics and statistics

To assist in kernel density estimation (KDE) and perform probability density calculations necessary to estimate the unknown distribution of the mutations found, the package Math.NET Numerics v3.2.3 [52] was used. The standard normal (Gaussian) kernel was used in the estimation. The equation used to estimate the probability density function induced by the mutations is as follows:

(2) $${\hat{f}}_{h}\left(x\right)=\frac{1}{nh}\sum_{i=1}^{n}K\left(\frac{x_{i}-x}{h}\right)$$

Where *x *is the point at which density is to be estimated, (*x*_1_, *x*_2_,..., *x_n_*) is the array of independent and identically distributed (i.i.d) sample (mutations) of some unknown distribution, *n *is the array size, *h *is the bandwidth, and *K *is the standard normal kernel function.

To account for the effect of copy number variation (CNV) on mutations found within regions containing copy number alterations, the following was done: i) copy number information was inferred using ExomeCNV for each loci of the tumor exome; ii) each mutation's loci was associated with those regions found in (i); iii) each mutation was weighted based on the copy number for the loci it lied within. The statistical software package R v3.1.3 [53], in particular, the KernSmooth (ks) package v1.9.4 [54] (hpi function) was used to calculate a data-derived univariate kernel density "plug-in" bandwidth [55]. The package R.NET v1.6 [56] was utilized to provide an application programming interface from .NET to R and the ks package.

#### Data storage, retrieval and annotation

At the Myeloma Institute the MPMDB is a pre-existing, research-driven molecular profiling repository. The design philosophy of the database was to provide a centralized information architecture for interfacing additional custom tools (e.g., iCloneViz) via various abstractions. The MPMDB provides many other features to facilitate the analysis of complex next generation sequencing (NGS) data, including extensive extract-transform-load (ETL) capabilities for data cleaning as well as data association and integration with various knowledge databases.

The integration of various modality datasets (aka tables or relations), which allow for the visualizations of iCloneViz are now described (N.B. not all attributes are defined for brevity). Standard database related theory and relational algebra notation are used [57]. A diagram titled, ***Multi-Omic Relational Integration ***(Additional File 1) illustrates the relations between the various relational algebra equations.

Let the schema of a relation be denoted as *A*(*a*_0_, *a*_1_, ..., *a_n_*), where *A *is the relation's name and each *a_i _*(0 ≤ *i *≤ *n*) is a name or identifier of an attribute within *A *(the domain or data type for each attribute *a_i _*are excluded for brevity ).

Let:

i. *W *be the resulting data from a whole exome sequencing experiment, that is, the SnpEff annotated results from the variant-calling application Strelka;

ii. *M *be the data resulting from a methylation experiment;

iii. *C *be the copy number data computationally inferred from a WES experiment using ExomeCNV;

iv. *H *be the raw count (unnormalized) data for each gene in an RNA-seq experiment using the htseq-count function from the HTSeq application;

v. *RV *be the data resulting from RNA-seq variant calling using the Haloptype Caller (GATK);

vi. *R *be RNA-seq transcript quantification data (FPKM) following transcriptome reconstruction by Cufflinks;

vii. *K *be a list of key genes representing cancer drivers and Variants of Uncertain Significance (VUS) the user is interested in labeling in each visualization; and

viii. *P *be patient and experimental meta-data.

The schema of each of the aforementioned relations to be integrated is defined as follows:

i. *W *(*Chromosome, Position, DNADepth, DNAAllelicFreq, EnsemblGeneId, GeneSymbol, DNAChange, AminoAcidChange, EffectImpact, ExperimenId*)

ii. *M *(*TargetId, AvgBeta, Annotation, EnsemblGeneId, ExperimentId*)

iii. C (*Chromosome, Start, Stop, CopyNumber, ExperimentId*)

iv. *H *(*EnsemblGeneId, Count, ExperimentId*)

v. *RV *(*Chromosome, Position, EnsemblGeneId, RNADepth, RNAAllelicFreq, ExperimentId*)

vi. *R *(*EnsemblTranscriptId, EnsemblGeneId, FPKM, ExperimentId*)

vii. *K*(*EnsemblGeneId*)

viii. *P *(*PatientId, FirstName, LastName, ExperimentDescription, ExperimentId, ExperimentDate, SampleType, SampleDescription, SampleCollectionDate*)

Let ⋈*_A.a=B.b _*denote the theta-join (in particular the equi-join) between relations *A *and *B *on attributes *a *and *b *respectively. Let ${⋈}_{L_{A.a=B.b}}$ denote the left-outer join between relations *A *and *B *on attributes *a *and *b *respectively. Let $\pi_{\left(a_{0},...,a_{n}\right)}$ denote the projection operator over attributes (*a*_0_,...,*a_n_*). Finally, let *σ_E_*(*A*) denote the selection operator, where *A *is the relation to be selected from and *E *is the conditional expression used for selecting tuples/rows from relation *A*. Given these definitions, and assuming each relation is selected for the experiment that is to be integrated, the final integration of the various multi-omic datasets can be defined as:

(3) $$I=\sigma_{\begin{array}{c} W.EffectImpact\\ {=}^{\prime }MODERATE^{\prime }\vee \\ W.EffectImpact\\ \;\;{=}^{\prime }HIGH^{\prime } \end{array}}\left(W{⋈}_{L_{=M.EnsemblGeneId}^{W.EnsemblGeneId}}M\right)$$

(4) $$J=\sigma_{R.FPKM>0}\left(RV{⋈}_{\begin{array}{c} RV.EnsemblGeneId\\ =R.EnsemblGeneId \end{array}}R\right)$$

(5) $$D=\sigma_{H.Count>0}\left(RV{⋈}_{\begin{array}{c} RV.EnsemblGeneId\\ =H.EnsemblGeneId \end{array}}H\right)$$

(6) $$L=I{⋈}_{\begin{array}{c} L\;W.Chromosome\\ =RV.Chromosome\wedge \\ \;\;W.Position\\ \;=RV.Position \end{array}}J$$

(7) $$N=L{⋈}_{\begin{array}{c} L\;W.Chromosome\\ =RV.Chromosome\wedge \\ \;\;W.Position\\ \;=RV.Position \end{array}}D$$

(8) $$IntegratedRelation=N{⋈}_{\begin{array}{c} L\;W.Chromosome\\ =C.Chromosome\wedge \\ \;W.Position\geq \\ \;\;C.Start\wedge \\ \;W.Position\\ \;\;\leq C.Stop \end{array}}C$$

Where *IntegratedRelation *denotes the final integrated relation. Given this final relation (and the key gene list *K *to visually label), aggregations and selections can be performed to filter data and encode all visual aspects within iCloneViz. Some of those aggregations and selections are now defined for later reference:

(9) $$W_{1}\cap_{\left(Chromosome,Position\right)}W_{2}$$

Where *W_i _*denotes *σ_W.ExperimentId=i_*(*W*) and ∩(*a*_0_,...,*a_n_*) is the intersection operator based on common attributes (*a*_0_,...,*a_n_*).

(10) $$W_{1}\backslash_{\left(Chromosome,Position\right)}W_{2}$$

Where \(*a*_0_,...,*a_n_*) denotes the set difference operator based on common attributes (*a*_0_,...,*a_n_*).

(11) $$Ŵ=distinct\left(\pi_{\begin{array}{c} Chromosome,\\ Position,\\ DNADepth,\\ CopyNumber,\\ DNAChange,\\ AminoAcidChange,\\ DNAAllelicFreq,\\ GeneSymbol,\\ Glyph \end{array}}\left(IntegratedRelation\right)\right)$$

Where *Glyph *= [1: *Only WES Data*, 2: *WES *+ *RNA Data*], is a computed attribute on *IntegratedRelation *indicating what type of data is available (not null) in each tuple of *IntegratedRelation*.

(12) $$\hat{R}\left(Ŵ\right)=distinct\left(\pi_{\begin{array}{c} \;GeneSymbol\\ EnsemblTranscriptId,\\ \;\;FPKM \end{array}}\left(\sigma_{\begin{array}{c} \;\;\;\;ŵ.Chromosome\\ =IntegratedRelation.Chromosome\\ \;\;\;\;\wedge ŵ.Position\\ =IntegratedRelation.Position \end{array}}(IntegratedRelation\right)\right)$$

Where  ŵ is a tuple within relation  Ŵ from Eq. (11).

(13) $$\hat{M}=distinct\left(\pi_{\begin{array}{c} \;TargetId,\\ \;AvgBeta,\\ GeneSymbol,\\ \;Annotation,\\ EnsemblGeneId,\\ \;\;Count \end{array}}\left(IntegratedRelation{⋈}_{\begin{array}{c} IR.EnsemblGeneId\\ =K.EnsemblGeneId \end{array}}K\right)\right)$$

(14) $$\hat{R}=distinct\left(\pi_{\begin{array}{c} \;GeneSymbol,\\ \;\;RNADepth,\\ RNAAllelicFreq,\\ \;\;FPKM,\\ EnsemblTranscriptId \end{array}}\left(IntegratedRelation\right)\right)$$

Pseudocode describing the algorithms of iCloneViz can be found in the appendix.

## Results and discussion

This study illustrates the integrated analysis of temporal-based clonal dynamics from a patient with MM. Examination of *Presentation *and *Relapse *samples from purified bone marrow aspirates are compared and contrasted using a bioinformatic approach named iCloneViz, which has been enhanced and redesigned to perform clonality analysis utilizing an integrative genomic (DNA, RNA, methylation) methodology, not previously possible. All graphs and visualizations in this study were generated by iCloneViz, unless indicated otherwise. It has been established that there is intraclonal heterogeneity at the level of single nucleotide variants (SNVs) in myeloma [58]. As a means to study the genetic heterogeneity and Darwinian nature that is germane to cancer, clonal analysis and integrative genomics has been advocated [59,60].

iCloneViz makes available a global view of all mutational events in a WES experiment. This is illustrated in Additional File 2. **Subfigure A **corresponds to the *Presentation *and **B **to the *Relapse *sample. The x-axis contains an ordered list of chromosomes (1-22, X, Y). Each chromosomal region is sized by the number of base pairs it contains. The y-axis is organized by variant allele frequency (VAF). Each variant is a point on the plot and the color scale indicates the depth of coverage (depth). A global view of mutations on a chromosomal basis is provided by these plots. A higher degree of mutations are evident on chromosomes eight and 14 for both *Presentation *and *Relapse*. Chromosome 18 contains more mutations in the *Relapse *sample vs. *Presentation*. Global views may provide rapid insights, especially as newly discovered types of complex genomic rearrangements, such as chromothripsis [61], katageis and the underlying roles for APOBEC and similar family enzymes [62], have their signatures or motifs become more understood.

A scatter plot of paired samples from *Presentation *and *Relapse *are profiled in Additional File 3. The x-axis contains the *Presentation *and y-axis the *Relapse*, and both axes are ordered by VAF. Key genes are labelled. Color discriminates paired mutations (blue) from those appearing only in the *Presentation *(green) and *Relapse *(red). A summary illustration of the mutational landscape is provided by this graphic, namely what is common and different between the two samples. There are a noteworthy fraction of shared mutations but also a reasonable amount of variants unique to each sample.

**Figure 1:**
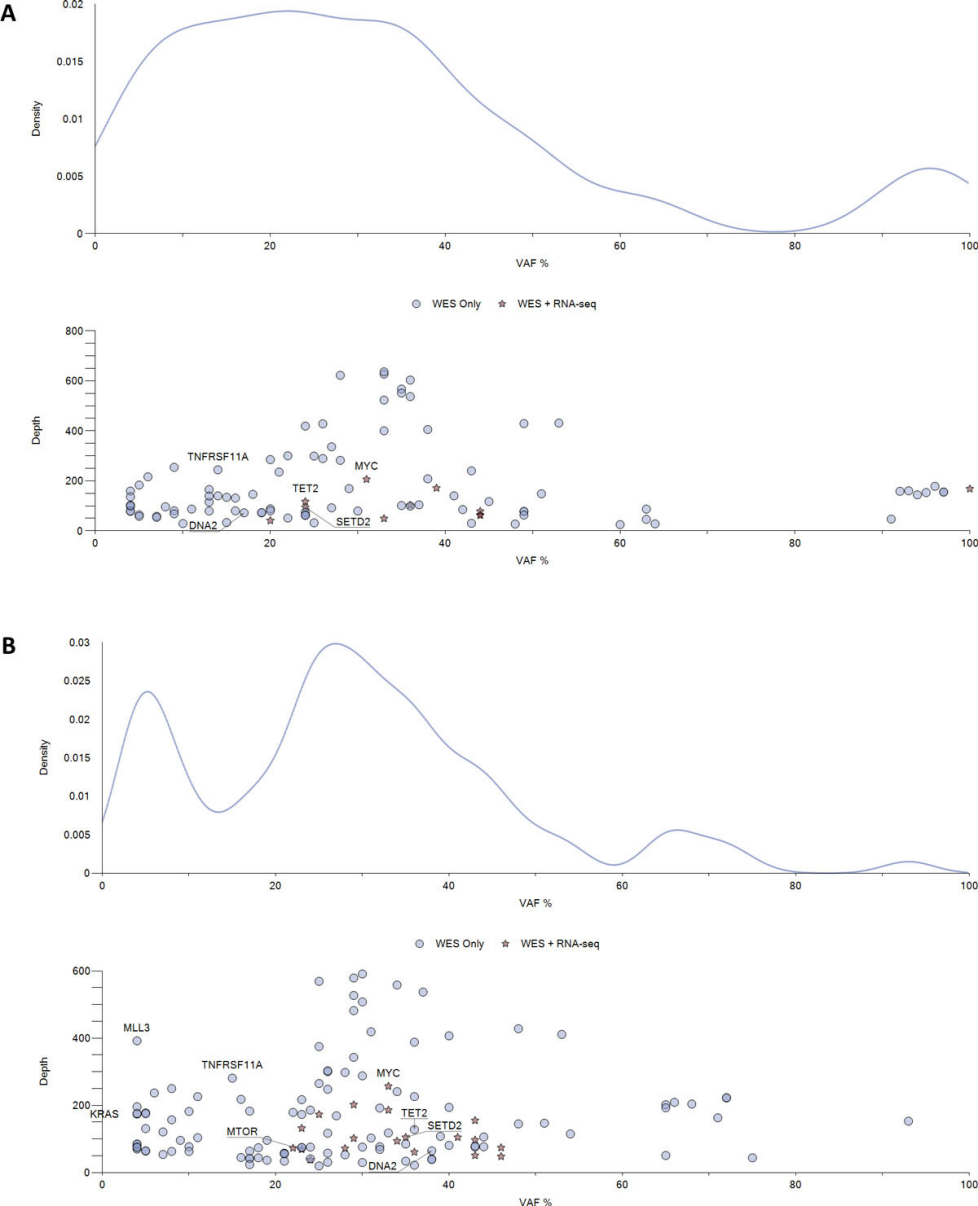
**Kernel density and scatter plot (all mutations)**. Gaussian kernel density plot and a paired scatter plot, for *Presentation *(**A**) and *Relapse *(**B**) samples, for all mutations found within the filtering criteria. The x-axis for all plots is the variant allele frequency (VAF). A Gaussian kernel density estimator is used to approximate the mutational density (weighted by copy number) of the mutations plotted in the upper subfigures. Lower subfigures (scatter plots) plot each mutation found (with key genes labelled). The y-axis of each lower subfigure is the depth of coverage. Mutations in the scatter plot are given a glyph that indicates the level of multi-omic data available for the given mutation, blue circle (WES only) and red star (WES and RNA-seq)

A visual exploration of integrated mutational data revealing the evolutionary trajectories is provided by iCloneViz. Figure 1 demonstrates Gaussian kernel density plots with paired scatter plots for the *Presentation *(**A**) and *Relapse *(**B**) samples. The paired plots serve to profile the magnitude and frequency of DNA mutations found in these tumor samples. The x-axis for each graph is VAF. The output of the kernel density function is contained on the y-axis. Peaks on the kernel density plots may infer dominant clones and subclones. A measure of relative abundance (Depth) [15] is contained on the y-axis of the scatter plot. Key genes are labelled, and two glyphs are used to signify if a mutation was found *only in *WES (blue circle) or found in *both *WES and RNA-seq (red star). A level of heterogeneity across the *Presentation *and *Relapse *samples is evident by the distinct peaks seen in the two kernel density plots.

**Figure 2:**
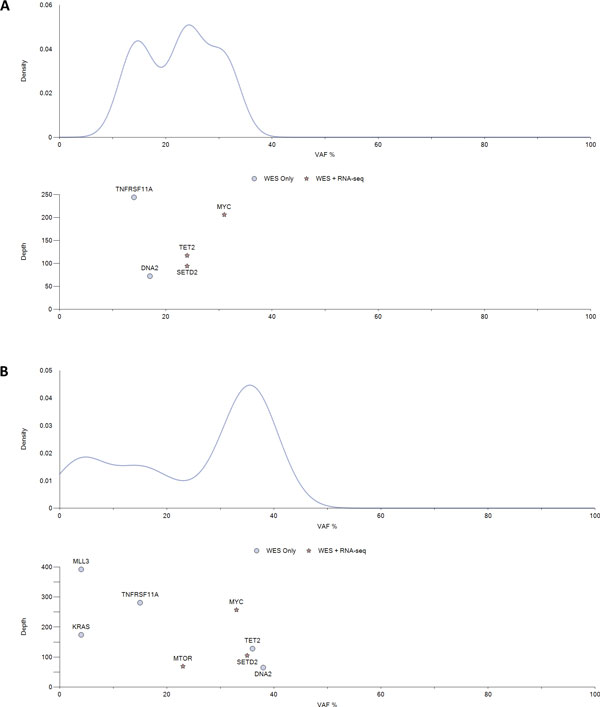
**Kernel density and scatter plot (key genes only)**. Gaussian kernel density plot and a paired scatter plot for *Presentation *(**A**) and *Relapse *(**B**) samples, showing key genes found within the filtering criteria only. The x-axis for all plots is the variant allele frequency (VAF). A Gaussian kernel density estimator is used to estimate the mutational density (weighted by copy number) of the mutations plotted in the upper subfigures. Lower subfigures (scatter plots) display each mutation found. The y-axis of each lower subfigure is the depth of coverage. Each mutation in the scatter plot are given a glyph that indicates the level of multi-omic data available for the given mutation. See legend for glyph assignments.

iCloneViz has a number of filtering options. Figure 2 shows a series of kernel density plots with associated scatter plots for the key genes found in the *Presentation *(**A**) and *Relapse *(**B**) samples. The units associated with the × and y axes are unchanged. The mutational glyph assignments are also without change. Distinct peaks are evident in both samples indicating heterogeneity.

**Figure 3:**
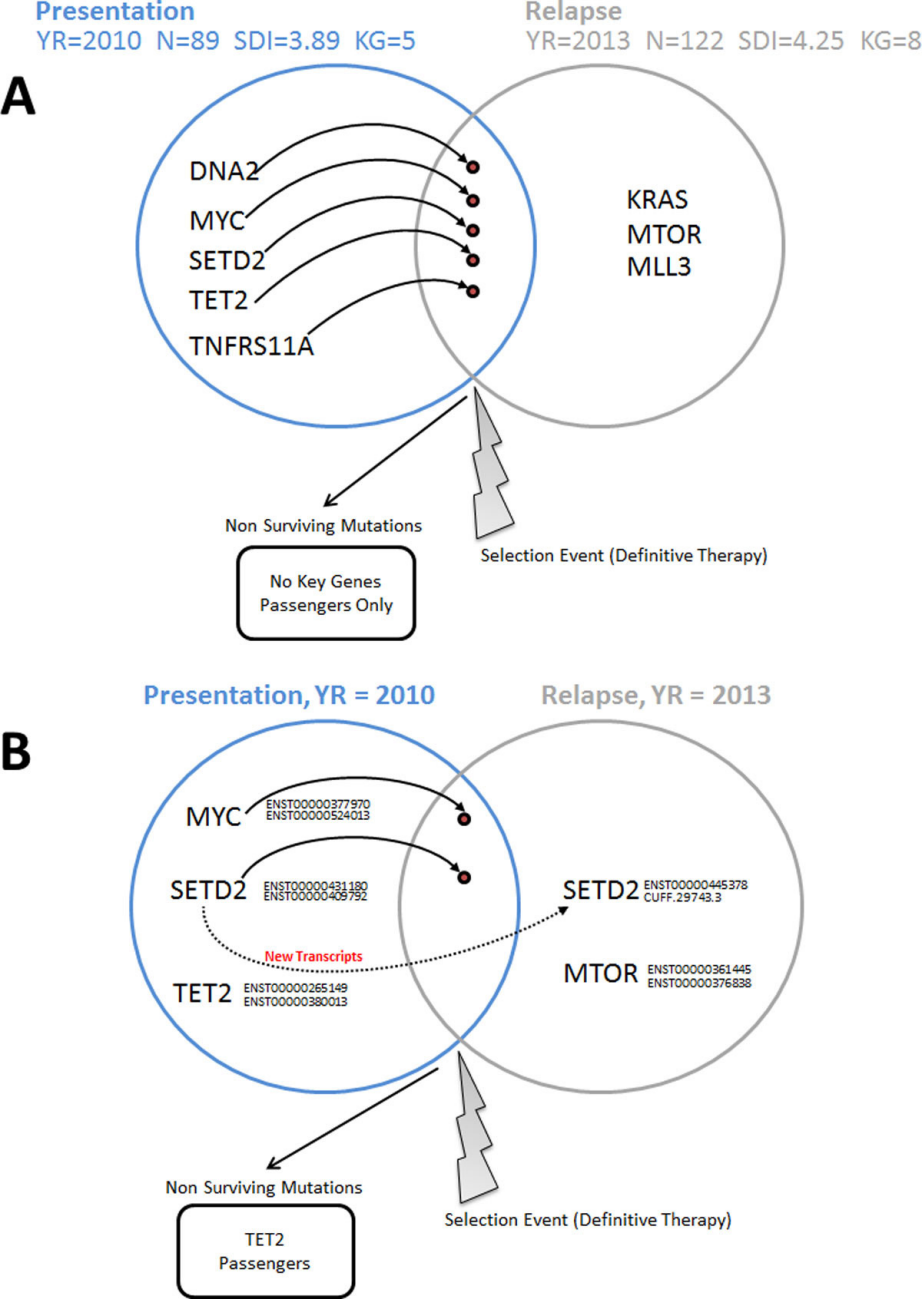
**DNA and RNA-based mutational dynamics and clonal evolution**. The temporal variant dynamics and clonal evolution of key genes across the *Presentation *(blue) and *Relapse *(grey) samples are illustrated. Subfigure **A **illustrates DNA level mutational dynamics and **B**, mRNA expressed mutations. Information regarding the year each sample was obtained, total number of mutations (N), computed Shannon Diversity Index (SDI), and the number of key genes (KG) are provided for each sample. Key genes identified within each sample are listed. An arc from a gene symbol into another sample indicates the survival of the mutation through the selection event (definitive therapy). Non-surviving key gene (as well as passengers) mutations are listed.

The temporal DNA-based mutational dynamics and clonal evolution observed in the *Presentation *and *Relapse *samples are shown in Figure 3A. This is an extension of the iCloneViz analysis. The *Presentation *sample was obtained when the patient was initially diagnosed with MM in year 2010. It contains 89 mutations that passed filtering criteria and was found to have a Shannon Diversity Index (SDI) of 3.89. There are five mutations classified from the key genes (KG) group and are: *DNA2, MYC, SETD2, TET2*, and *TNFRS11A*.

An evolutionary selection event occurred later in year 2010, as a result of the patient receiving definitive therapy for MM. The patient did well until year 2013 when a relapse occurred and a new bone marrow aspirate was obtained. Comparing the *Presentation *to *Relapse *reveals that all the key genes survive, including the amplified *MYC *oncogene, which also contains a VUS missense mutation. In addition three more mutated genes are gained including the histone methyltransferase *MLL3*, along with the *KRAS *and *MTOR *oncogenes.

A higher number of mutations (122) are found in the *Relapse *sample. It also has an increased SDI (4.25), which implies more uncertainty or randomness in the process, from an information theory viewpoint [45]. From a cancer biology view, it indicates a progression of disease or diversification in the mutational landscape.

Table 1 lists the summary information for the *Presentation *key genes, and Table 2 for the *Relapse *key genes. Columns are *Gene *for gene symbol, *DNA Mutation, AA Change *for the corresponding amino acid change, *CN *for copy number, *DP *for depth, *AF *for allelic frequency, *RNA Mutation *to indicate expressed mutations, and *Notes *that contains the gene class. Noted are three mutations in the *Presentation *group (*MYC, SETD2, TET2*), which have their DNA mutations also found to be expressed in mRNA. The Relapse key genes also contain three DNA variants found to have their mutations expressed in RNA, and include the still amplified and mutated *MYC *oncogene, *SETD2*, and the *MTOR *oncogene that contains a hotspot mutation (p.Val2006Leu).

**Table 1:** DNA-based *Presentation *key genes

Gene	DNA Mutation	AA Change	CN	DP	AF	RNA Mutation	Notes
DNA2	c.146delG		1	82	17	N	DNA repair

MYC	c.226G>A	p.Ala76Thr	3	207	31	Y	Amp oncogene + VUS

SETD2	c.7085A>G	p.Gln2362Arg	1	94	24	Y	HMT

TET2	c.2725C>T	p.Gln909*	3	117	24	Y	Oncogene

TNFRSF11A	c.1097C>T	p.Pro366Leu	3	244	14	N	Bone remodelling

A tabular version of the clonal dynamics displayed in Figure 3A lists changes in allelic frequency, depth and copy number for the *Presentation *sample. Also indicated is whether or not the DNA mutation was expressed in the sample's associated mRNA. Abbreviations: CN (Copy Number), DP (Depth), Allelic Frequency (AF), VUS (Variant of Uncertain Significance), HMT (Histone Methyltransferase), AA (Amino Acid) and Amp (Amplified).

**Table 2:** DNA-based *Relapse *key genes

Gene	DNA Mutation	AA Change	CN	DP	AF	RNA Mutation	Notes
DNA2	Same	same	3	70	38	N	

MYC	Same	same	3	257	33	Y	

SETD2	Same	same	2	105	35	Y	

TET2	Same	same	3	129	36	N	

TNFRSF11A	Same	same	3	283	15	N	

KRAS	c.183A>C	p.Gln61His	3	175	4	N	Oncogene

MTOR	c.6016G>C	p.Val2006Leu	1	69	23	Y	Oncogene

MLL3	c.944G>A	p.Gly315Asp	1	392	4	N	HMT

A tabular version of the clonal dynamics showed in Figure 3A lists changes in allelic frequency, depth and copy number for the *Relapse *sample.

Figure 3B illustrates the temporal RNA-based mutational dynamics and clonal evolution. This is also an extension of the iCloneViz analysis. The *MYC *oncogene and *SETD2*, in addition to having their DNA mutations found in mRNA, have two transcripts in the *Presentation *that are also found in the *Relapse *sample. *SETD2 *also has two new transcripts appearing in the *Relapse *sample. CUFF.29743.3 is a novel transcript due to a new exon. However, this exon was found to have only one supporting read by IGV, and thus not seriously considered to be viable. The *TET2 *transcripts do not survive and are listed in the "Non Surviving Mutations" box along with passengers. *MTOR*, which was a new DNA mutation found in the *Relapse *sample, has two viable mRNA transcripts now appearing in the *Relapse*. Table 3 lists the expressed RNA mutated key gene transcripts for the *Presentation *sample and Table 4 for *Relapse*.

**Table 3:** mRNA expression of key gene mutations in the *Presentation *sample

Gene	DP	AF	Transcript	FPKM
MYC	192	100	ENST00000377970	42

MYC	192	100	ENST00000524013	58

SETD2	25	50	ENST00000409792	9.5

SETD2	25	50	ENST00000431180	2.2

TET2	6	50	ENST00000380013	2.0

TET2	6	50	ENST00000265149	1.2

A tabular version of the clonal dynamics as shown in Figure 3B, lists changes in depth, allelic frequency and FPKM in the *Presentation *sample. Abbreviations: DP (Depth) and Allelic Frequency (AF).

**Table 4:** mRNA expression of key gene mutations in the *Relapse *sample

Gene	DP	AF	Transcript	FPKM
MYC	100	100	ENST00000377970	24

MYC	100	100	ENST00000524013	55

SETD2	29	50	ENST00000409792	3.2

SETD2	29	50	ENST00000431180	1.5

SETD2	29	50	ENST00000445387	1.2

SETD2	29	50	CUFF.29743.3	9.4

MTOR	20	50	ENST00000361445	4.7

MTOR	20	50	ENST00000376838	2.2

A tabular version of the clonal dynamics as shown in Figure 3B, lists changes in depth, allelic frequency and FPKM for the *Relapse *sample.

Table 5 lists the DNA methylation of tumor suppressor genes (TSGs) for the *Presentation *sample passing iCloneViz filtering criteria from ~485,000 possible candidates. The filtering criteria consisted of: **i**) Avg-Beta ≥ 25%, **ii**) "*Promoter Associated*" regulatory feature, and **iii**) being from a CpG Island. The methylation data set was integrated with RNA-seq to directly associate the raw (unnormalized) *RNA Read Counts *for the TSGs identified by filtering criteria. The majority of TSGs in the *Presentation *(Table 5) and *Relapse *(Table 6) have zero or very low read counts, indicating a lack of mRNA expression and thus no protein production. Loss of TSG(s) is associated with both the onset and progression of many cancers [63,64].

**Table 5:** DNA methylation of TSGs in the *Presentation *sample

Target ID	Avg-Beta	Gene	Ensembl GeneID	RNA Read Count	Classification	Regulatory Feature	Relation to CpG Island
cg27549619	0.3934072	AXIN1	ENSG00000103126	4	TSG	Promoter Associated	Island

cg26370022	0.6804651	BRCA1	ENSG00000012048	0	TSG	Promoter Associated	Island

cg11529738	0.7789396	BRCA1	ENSG00000012048	0	TSG	Promoter Associated	Island

cg24900425	0.9020266	BRCA1	ENSG00000012048	0	TSG	Promoter Associated	Island

cg13601799	0.2905587	CDKN2A	ENSG00000147889	13	TSG	Promoter Associated	Island

cg01437571	0.3243266	CEBPA	ENSG00000245848	0	TSG	Promoter Associated	Island

cg00976692	0.291538	MEN1	ENSG00000133895	8	TSG	Promoter Associated	Island

cg14803009	0.3387372	MSH2	ENSG00000095002	0	TSG	Promoter Associated	Island

cg22866426	0.4628801	RUNX1	ENSG00000159216	1	TSG	Promoter Associated	Island

cg27003951	0.2562254	SOCS1	ENSG00000185338	0	TSG	Promoter Associated	Island

Listed are the DNA methylation of tumor suppressor genes (TSGs) passing iCloneViz filtering criteria from ~485,000 possible candidates, for the *Presentation *sample. The filtering criteria consisted of: **i**) Avg-Beta ≥ 25%, **ii**) "*Promoter Associated*" regulatory feature, and **iii**) being from a CpG Island. The methylation data set was integrated with RNA-seq to directly associate the raw (unnormalized) *RNA Read Counts *for the identified TSGs.

**Table 6:** DNA methylation of TSGs in the *Relapse *sample

Target ID	Avg-Beta	Gene	Ensembl Gene ID	RNA Read Count	Classification	Regulatory Feature	Relation to CpG Island
cg27549619	0.443731	AXIN1	ENSG00000103126	0	TSG	Promoter Associated	Island

cg02086790	0.4921295	AXIN1	ENSG00000103126	0	TSG	Promoter Associated	Island

cg26370022	0.6747859	BRCA1	ENSG00000012048	0	TSG	Promoter Associated	Island

cg24900425	0.8910863	BRCA1	ENSG00000012048	0	TSG	Promoter Associated	Island

cg11529738	0.7637555	BRCA1	ENSG00000012048	0	TSG	Promoter Associated	Island

cg13601799	0.467826	CDKN2A	ENSG00000147889	38	TSG	Promoter Associated	Island

cg00976692	0.293402	MEN1	ENSG00000133895	0	TSG	Promoter Associated	Island

cg14803009	0.4267139	MSH2	ENSG00000095002	0	TSG	Promoter Associated	Island

cg22073802	0.2875548	PTCH1	ENSG00000185920	23	TSG	Promoter Associated	Island

cg22866426	0.5251715	RUNX1	ENSG00000159216	0	TSG	Promoter Associated	Island

cg17339910	0.399428	RUNX1	ENSG00000159216	0	TSG	Promoter Associated	Island

cg04004558	0.2809271	SOCS1	ENSG00000185338	0	TSG	Promoter Associated	Island

cg27003951	0.2598022	SOCS1	ENSG00000185338	0	TSG	Promoter Associated	Island

Listed are the DNA methylation of tumor suppressor genes (TSGs) passing iCloneViz filtering criteria from ~485,000 possible candidates, for the *Relapse *sample. Filtering criteria and integration with RNA-seq is the same as is reported for the *Presentation *sample (Table 5).

Why did the patient's cancer recur following definitive therapy? Does the experimental information from DNA, RNA, and methylation analyses provide answers or insights concerning the relapse? Regarding the *Presentation *sample, associating the findings from Figure 1A, and Figure 2A, the founder clone contains an amplified and mutated *MYC *oncogene. Table 1 reports a missense mutation in *MYC *having the base substitution of c.226G>A, resulting in alanine being replace by threonine at position 76 (p.Ala76Thr) in the amino acid chain. The copy number is 3 (amplified), depth of coverage is 207 and the allelic fraction is 31. This mutation is also expressed in mRNA. Additional File 4 shows a *MYC *lolliplot diagram made by the Protein Paint [65] application showing the possible missense mutations, depending on splicing, in the coding region. Table 3 shows the *MYC *transcripts with good depth of coverage and FPKMs. Finally, Table 5 shows four TSGs (*BRCA1, CEBPA, MSH2, SOCS1*) with a CpG Island, promoter associated regulatory feature being methylated by more than 25% and having RNA read counts of zero, indicating these genes have been silenced.

Concerning the *Relapse *sample, integrating findings from Figure 1, **B **and Figure 2, **B **shows that the founder clone continues to contain the mutated *MYC *oncogene. Table 2 reports the same *MYC *DNA mutation, the copy number is still amplified at three, DP is 257, allelic frequency is 33%, and the mutation is present in mRNA. The *MTOR *oncogene is new, and contains a hotspot mutation (c.6016G>C, p.Val2006Leu), which is also found in RNA. Figure 3B shows survival of *MYC *related transcripts from *Presentation *to *Relapse*, and the transcripts for *MTOR*. Table 4 shows the two *MYC *transcripts with good depth of coverage and FPKMs, and the *MTOR *transcripts have reasonable values. Table 6 displays six TSGs (*AXIN1, BRCA1, MEN1, MSH2, RUNX1, SOCS1*) with CpG Island promoter associated regulatory feature being methylated by more than 25% and having RNA read counts of zero, indicating a progression of gene silencing. The *KRAS *oncogene appears in the Relapse but the mutation was not expressed in RNA (Table 2) therefore would not be considered for therapeutic targeting.

Deregulated expression of *MYC *is a hallmark feature of cancer and serves to uncouple growth factor dependent proliferation [66], and may occur through a variety of mechanisms (e.g., gene amplification, translocation, focal enhancer amplification, or constitutive activation of upstream signalling pathways) [67]. *MYC *over-expression occurs in ~30% of human cancers and commonly is a harbinger for a poor clinical outcome, aggressive biological behavior, increased chance of relapse and advanced stage of disease at initial diagnosis [68]. Studies in transgenic mouse models have identified *MYC *inactivation leads to prompt tumor regressions [69].

There are now a number of new agents in clinical trials for targeting *MYC*, for instance the BET bromodomain inhibition [70,71], and this should be considered for subsequent therapy. Additionally, the *Relapse *sample contained a hotspot mutation in *MTOR *and targeting with a "rapalog" (rapamycin and its analogs) should also be considered. Precision medicine and therapeutic combinations with new and more targeted agents are challenging and an active area in clinical trials and translational research [72,73]. Essential to these efforts are advanced bioinformatics with abilities to integrate multi-omic datasets, combat cancer heterogeneity via clonal and evolutionary approaches, and ultimately provide clinical utility thru an improved understanding of the disease process at hand. This was demonstrated in this study.

## Conclusions

Illustrated in this study has been the temporal-based analysis of integrated clonal dynamics of a single patient with MM by examining the *Presentation *and *Relapse *tumor samples. This involved the visualization and quantification of variant/mutational dynamics in the context of *integrated evolution *of WES, RNA-seq, and DNA methylation. Subpopulations of mutations will evolve over time due to natural selection events related to cell intrinsic or micro-environmental factors, as well as selection events induced therapeutically. Selection events eliminate some mutations and provide a survival advantage to others. iCloneViz provides global views of mutational events as well as rapid data integration of **i**) WES, **ii) **RNA-seq, and **iii**) DNA methylation. This results in an enriched picture with a more focused specificity of mutational events, and provides evidence and more confidence for therapeutic assignments.

Heterogeneity is found in many cancers and limits aggregate approaches for scientific and clinical utility. MM is known as a heterogeneous cancer with a complex molecular landscape. Clonality and integrated genomics has been advocated as means to combat heterogeneity [59,60]. The illustrated *novelty and precise contribution *of iCloneViz is the ability to perform integrative clonality analysis utilizing data sets from: **i**) WES, **ii**) RNA-seq, and **iii**) DNA methylation. To date, there is no other software based clonality tool available (commercially or open source) that can perform integrative clonality analysis. Given the repeated findings of multiple TCGA studies reporting heterogeneity with a complex mutational landscape [14,74], additional analytical approaches are needed; since it is evident that DNA analysis is **necessary but not sufficient**. Our approach, which builds on previous work now employs clonality and integrative genomics, both of which have been recommended to combat heterogeneity.

In this study, observed in all serial MM samples was the presence of an amplified *MYC *oncogene species with a VUS missense mutation. *MYC *is a transcription factor and master regulator of ~15% of all gene expression, and also functions to regulate chromatin structure. A permanent remission or cure was not achieved despite definitive therapy. The dominant genetic alterations in the founder clone was never targeted specifically for therapy thus cure or a lasting remission was unlikely. For major advances in cancer management, a systematic approach to collect tissue samples at diagnosis, and serially at relapse(s), in order to profile the dynamic clonal evolution is critical.

## Appendix

### iCloneViz pseudocode

The pseudocode for iCloneViz is now presented, and is described and documented using a PDL (Program Design Language) structure as described in Pressman [75]. A C-style notation was utilized. A flow diagram of the pseudocode is shown in Additional File 5. The multi-omic relational integration is illustrated in Additional File 1.

Symbol Definitions:

● patient_id: Patient identifier.

● button_selection: An instance of the button that was clicked.

● button_click_filter: Boolean indicating whether an instance of the button labeled "Filter" was clicked.

● button_click_show_tsg_methylation_table: Boolean indicating whether an instance of the button labeled "Show TSG Methylation Table" was clicked.

● button_click_show_wes_table: Boolean indicating whether an instance of the button labeled "Show WES Table" was clicked.

● button_click_show_rna_table: Boolean indicating whether an instance of the button labeled "Show RNA Table" was clicked.

● exp_id []: Array of one or two patient experiment identifiers. Can be referenced via subscripting (e.g., exp_id [0]).

● exp_id[i].W: WES data in relation *W *for the patient experiment with the experiment identifier exp_id[i].

● filter_settings: Data structure containing all filter settings including:

○ min_vaf: Minimum variant allele frequency (default 4%).

○ max_vaf: Maximum variant allele frequency (default 100%).

○ min_depth: Minimum read depth (default 20).

○ max_depth: Maximum read depth (default 1000).

○ min_meth: Minimum methylation percent (default 25%).

○ opacity: Opacity of scatter plot points (default 50%).

○ show_kg_only: Boolean indicating whether to show only mutations found in the key genes list *K *(default False).

● default_filter_settings: Default values used for filtering.

/*******************************************************

Name & Purpose: Main, program entry point

Inputs: None

Processing: Establish main processing loop for iCloneViz

Outputs: None

Returns: None

Authors: D. Johann, E. Peterson

********************************************************/

main()

{

while (window is open)

{

patient_id = display_patient_search();

button_selection, exp_id[] = display_patient_experiments(patient_id);

process(button_selection, exp_id[]);

}

}

/*******************************************************

Name & Purpose: Display Patient Search, via patient ID display patient meta-data

Inputs: none

Processing: Display patient meta-data

Outputs: None

Returns: ID of selected patient from MPMDB

Authors: D. Johann, E. Peterson

********************************************************/

display_patient_search()

{

patient_id = input from user;

- get / display patient meta-data via relation *P*;

return patient_id;

}

/*******************************************************

Name & Purpose: Display Patient Experiments, show available experiments for iCloneViz analysis

Inputs: Patient ID

Processing: Retrieve patient experimental meta-data from MPMDB

Outputs: Patient experimental data now in memory

Returns: Array of experiment IDs, Button selection for selected analysis, eg, Genomic Real Estate or Paired Scatter Plot or KDE plus Scatter Plot

Authors: D. Johann, E. Peterson

********************************************************/

display_patient_experiments(patient_id)

{

- get / display all experimental data from database (MPMDB) for patient via relation *P*;

exp_id[] = selected experiment ids;

return button_selection, exp_id[]

}

/*******************************************************

Name & Purpose: Process, process data and display visualization based on the user's choice of visualization

Inputs: Button selection, & Experiment IDs

Processing: Execute specific function to handle processing based on user's choice of visualization

Outputs: None

Returns: None

Authors: D. Johann, E. Peterson

********************************************************/

process(button_selection, exp_id[])

{

if (button_selection == 'Genomic Real Estate')

genomic_real_estate(exp_id[0]);

else if (button_selection == 'Paired Scatter Plot')

paired_scatter_plot(exp_id[0...1]);

else if (button_selection == 'KD + Scatter Plot')

kd_plus_scatter_plot(exp_id[0]);

}

/*******************************************************

Name & Purpose: Genomic Real Estate, fetch WES-based mutation data, using R.NET to generate R plot and visualize

Inputs: Experiment ID of experiment to visualize

Processing: Using R.NET API, generate plot image and display in window

Outputs: Scatter plot of all mutations by chromosome and variant allele frequency, read depth is encoded by color, see Additional File 2

Returns: None

Authors: D. Johann, E. Peterson

********************************************************/

genomic_real_estate(exp_id)

{

- execute query to MPMDB to fetch mutation data based on the exp_id and DB relation *W*;

- establish the R.NET interface and invoke R;

- divide x-axis into 24 sections (22 chromosome + X,Y)

- scale each section by the length of each chromosome

for each *w *in *W*

{

- plot *w *along x-axis by position, the y-axis by variant allele frequency, and color by read depth;

}

- export plot as image file;

- return execution to .NET;

- display image file in windows form;

}

/*******************************************************

Name & Purpose: Paired Scatter Plot, fetch filter settings and call function to display paired scatter plot

Inputs: Experiment IDs

Processing: If first time displaying, use default filter settings to display paired plot, otherwise, fetch filter settings from user and display paired plot

Outputs: None

Returns: None

Authors: D. Johann, E. Peterson

********************************************************/

paired_scatter_plot(exp_id[])

{

display_paired_scatter_plot(default_filter_settings, exp_id[]);

while (window is open)

{

filter_settings = read_filter_toolbar();

display_paired_scatter_plot(filter_settings, exp_id[]);

}

}

/*******************************************************

Name & Purpose: Display Paired Scatter Plot, calculate WES mutations in common and exclusive to each experiment and generate paired scatter plot

Inputs: Filter settings fetched from user input, and Experiment IDs

Processing: Fetch WES mutations, and using filter settings and referenced equations, calculate mutations in common and exclusive to each experiment; display paired scatter plot with data, see Additional File 3

Outputs: A paired scatter plot for each experiment in exp_id array, based on variant allele frequency

Returns: None

Authors: D. Johann, E. Peterson

********************************************************/

display_paired_scatter_plot(filter_settings, exp_id[])

{

// Relational algebra Eq. (9)

common = exp_id[0].W ∩ exp_id[1].W;

// Relational algebra Eq. (10)

exp0_unique = exp_id[0].W \ exp_id[1].W;

// Relational algebra Eq. (10)

exp1_unique = exp_id[1].W \ exp_id[0].W;

- label all genes in *K*;

if (filter_settings.show_kg_only == true)

- hide all non-labelled points;

- plot common, exp0_unique, and exp1_unique based on DNAllelicFreq (variant allelic frequency) using filter-settings;

}

/*******************************************************

Name & Purpose: Read Filter Toolbar, gather user-defined filter settings, and display "TSG Methylation Table", "WES Table", or "RNA Table" if the user so desires

Inputs: None

Processing: Collect filter settings from user; if the user clicks on "Filter" the filter settings are returned and they are used when displaying a desired plot; if the user clicks on "Show TSG Methylation Table", "Show WES Table", or "Show RNA Table", the desired table is displayed using the referenced equations

Outputs: Tables selected if clicked

Returns: User-defined filter settings

Authors: D. Johann, E. Peterson

********************************************************/

read_filter_toolbar()

{

while (true)

{

filter_settings.min_vaf = user input minimum VAF;

filter_settings.max_vaf = user input maximum VAF;

filter_settings.min_depth = user input minimum read depth;

filter_settings.max_depth = user input maximum read depth;

filter_settings.min_meth = user input minimum methylation percent;

filter_settings.opacity = user input scatter plot point opacity;

filter_settings.show_kg_only = user input show key gene only;

if (button_click_filter)

  return filter_settings;

else if (button_click_show_tsg_methylation_table)

  - display TGS Methylation Table via Relational algebra Eq. (13);

  break;

else if (button_click_show_wes_table)

  - display WES Table Eq. (11);

  break;

else if (button_click_show_rna_table)

  - display RNA Table Eq. (14);

  break;

}

return filter_settings;

}

/*******************************************************

Name & Purpose: KD Plus Scatter Plot, call subroutines to process and render data for each of the individual plot areas, as well as, to calculate various metircs

Inputs: Experiment ID of experiment to visualize

Processing: Call subroutines to process and render the individual plot areas; call subroutine to calculate various metrics

Outputs: None

Returns: None

Authors: D. Johann, E. Peterson

********************************************************/

kd_plus_scatter_plot(exp_id)

{

display_kd_plot(default_filter_settings, exp_id);

display_scatter_plot(default_filter_settings, exp_id);

calculate_metrics(default_filter_settings, exp_id);

while (window is open)

{

filter_settings = read_filter_toolbar();

display_kd_plot(filter_settings, exp_id);

display_scatter_plot(filter_settings, exp_id);

calculate_metrics(filter_settings, exp_id);

}

}

/*******************************************************

Name & Purpose: Display KD Plot, displays the kernel density estimation curve, using the 'ks' R package to calculate an appropriate bandwidth

Inputs: User-defined filter settings and the experiment ID to be visualized

Processing: Calculate the KDE for DNA mutations, use R.NET to utilize the 'ks' package (used for bandwidth calculation)

Outputs: KD curve, see Figures 1 & 2

Returns: None

Authors: D. Johann, E. Peterson

********************************************************/

display_kd_plot(filter_settings, exp_id)

{

- calculate KDE based on Relational algebra Eq. (11) and Eq. (2) for all mutations in Ŵ and weighted by copy number;

- utilize R.NET to call 'hpi' function in the R 'ks', for bandwidth calculation;

- display KD plot using filter_settings;

}

/*******************************************************

Name & Purpose: Display Scatter Plot, displays the DNA mutational scatter plot, and tooltip containing RNA expression based info if available

Inputs: User-defined filter settings and the experiment ID to be visualized

Processing: Displays a scatter plot point for each DNA mutation, set each glyph depending on the degree of modality data available, and populate tooltip with RNA expression data if available

Outputs: Mutational scatter plot, see Figures 1 & 2

Returns: None

Authors: D. Johann, E. Peterson

********************************************************/

display_scatter_plot(filter_settings, exp_id)

{

- calculate Ŵ by Relational algebra Eq. (11) using filter_settings;

- set glyph for all DNA mutations to be a blue circle and place along a-axis according to variant allele frequency and along y-axis by depth

for each tuple ŵ in Ŵ having RNA data calculate $\hat{R}\left(ŵ\right)$ by Relational algebra Eq. (12)

{

- update point glyph, (red star, expressed RNA mutation);

- build hover-over tooltip to contain: gene name, transcript(s) ids and FPKM(s) from $\hat{R}\left(ŵ\right)$;

}

- display scatter plot for each mutation in Ŵ with associated tooltip (if RNA data is available);

}

/*******************************************************

Name & Purpose: Calculate Metrics, calculate various metrics for display

Inputs: User-defined filter settings and the experiment ID to be visualized

Processing: Calculate SDI, total number of mutations, and total number of key gene mutations, for the data being visualized

Outputs: Calculated metrics

Returns: None

Authors: D. Johann, E. Peterson

********************************************************/

calculate_metrics(filter_settings, exp_id)

{

- calculate SDI using Eq. (1) and relation Ŵ (Relational algebra Eq. (11)) using filter_settings;

- calculate total number of mutations in relation Ŵ using filter_settings;

- calculate total number of key genes from *K *which are found in relation Ŵ using filter_settings;

- display metrics;

}

## Competing interests

The authors declare that they have no competing interests.

## Authors' contributions

DJJ and EAP conceived and designed the study. EAP, MAB, SSC, CJH, NW and DJJ performed experiments and analyses. DJJ and EAP designed the software. EAP, DJJ and MAB implemented the software. DJJ and EAP wrote the manuscript. All authors approved the manuscript.

## Supplementary Material

Additional File 1 **Muti-omic relational integration**. Illustration of relations used to integrate multi-omic datasets. The upper-right box (Relations & Attributes) defines all relations and their attributes. The left-most section (iCloneViz DB) lists each dataset and a name / identifier for each. Middle section (Multi-Omic Integration) illustrates the relationships and integration of each dataset using intermediate relations. Each intermediate integration is annotated with the attributes used in each combination. Each intermediate relation is further annotated with the equation used in the manuscript to form the given relation. The final multi-omic "Integrated Relation" is shown in the lower right. Click here for file

Additional File 2 Genomic mutational overview. A genomic mutational overview of two experiments is computed and displayed. **A **corresponds to the *Presentation *sample and **B **to *Relapse*. These provide a general view of the inherent mutational events on a chromosomal basis. The x-axis contains an ordered list of chromosomes (1-22, X, Y), each sized by the number of base pairs (bp) it contains. The y-axis is ordered by variant allele frequency (VAF), and the color scale indicates sequence depth. Each variant is a point in the plot. Click here for file

Additional File 3 Scatter plot of paired samples. Displayed are variants in the *Presentation *on the x-axis compared to *Relapse *on y-axis. Both the × and y-axes are based on VAF. Variants are colored to indicate whether they are shared or unique. See legend for color assignments. Click here for file

Additional File 4 **MYC oncogene with mutation showing possible splicing events**. Illustrated is a lolliplot diagram of MYC showing the possible missense mutations in the coding region depending on splicing. Click here for file

Additional File 5 **iCloneViz pseudocode flow diagram**. Illustrated is the execution flow of iCloneViz and its associated subroutines. Each subroutine and its formal parameters is represented as a node. Each arc represents a subroutine call from one subroutine to another. Click here for file

## Declarations

Publication of this work was supported by Program Project Grant CA 55813, from the National Institutes of Health (NIH), National Cancer Institute (NCI), Bethesda, MD, USA.

This article has been published as part of *BMC Bioinformatics *Volume 16 Supplement 13, 2015: Proceedings of the 12th Annual MCBIOS Conference. The full contents of the supplement are available online at http://www.biomedcentral.com/bmcbioinformatics/supplements/16/S13.
