# Supplementary figures and images for: Enhancing cancer clonality analysis with integrative genomics
Source: BMC Bioinformatics. 2015 Sep 25;16(Suppl 13):S7. doi: 10.1186/1471-2105-16-S13-S7 (PMC4597064; doi:10.1186/1471-2105-16-S13-S7)

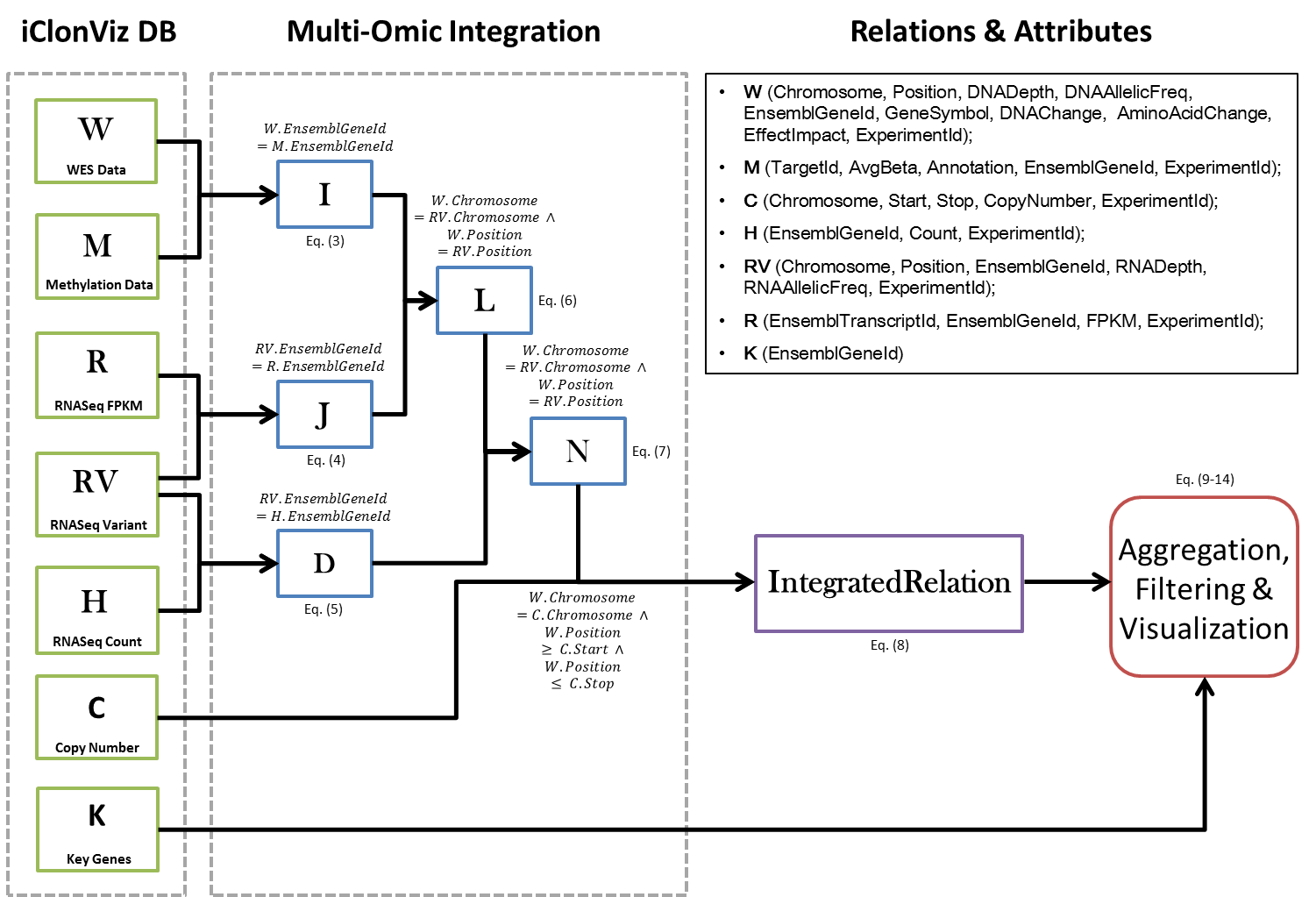

Supplement: Additional File 1 — Muti-omic relational integration. Illustration of relations used to integrate multi-omic datasets. The upper-right box (Relations & Attributes) defines all relations and their attributes. The left-most section (iCloneViz DB) lists each dataset and a name / identifier for each. Middle section (Multi-Omic Integration) illustrates the relationships and integration of each dataset using intermediate relations. Each intermediate integration is annotated with the attributes used in each combination. Each intermediate relation is further annotated with the equation used in the manuscript to form the given relation. The final multi-omic "Integrated Relation" is shown in the lower right. [file 1471-2105-16-S13-S7-S1.png]

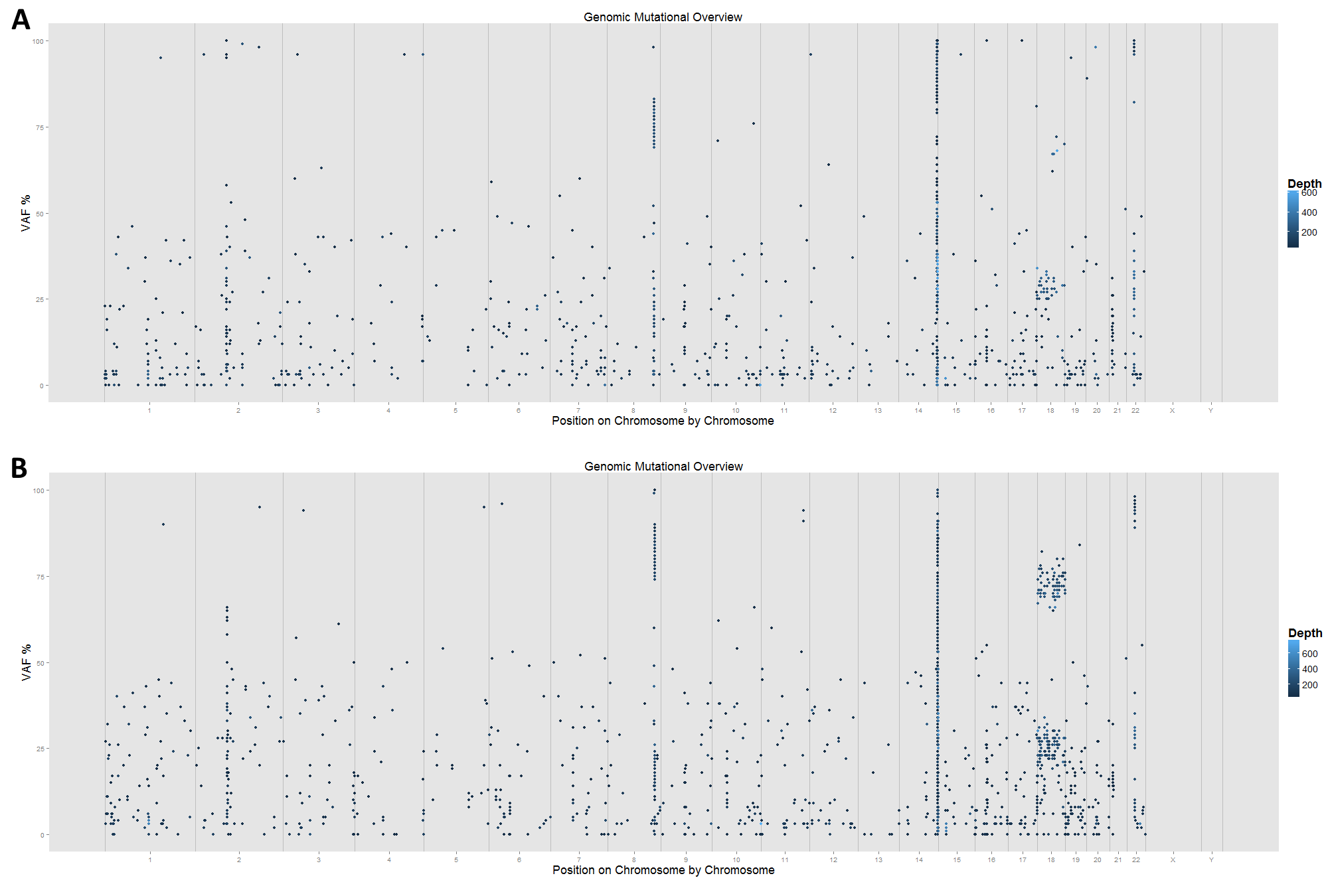

Supplement: Additional File 2 — Genomic mutational overview. A genomic mutational overview of two experiments is computed and displayed. A corresponds to the Presentation sample and B to Relapse. These provide a general view of the inherent mutational events on a chromosomal basis. The x-axis contains an ordered list of chromosomes (1-22, X, Y), each sized by the number of base pairs (bp) it contains. The y-axis is ordered by variant allele frequency (VAF), and the color scale indicates sequence depth. Each variant is a point in the plot. [file 1471-2105-16-S13-S7-S2.png]

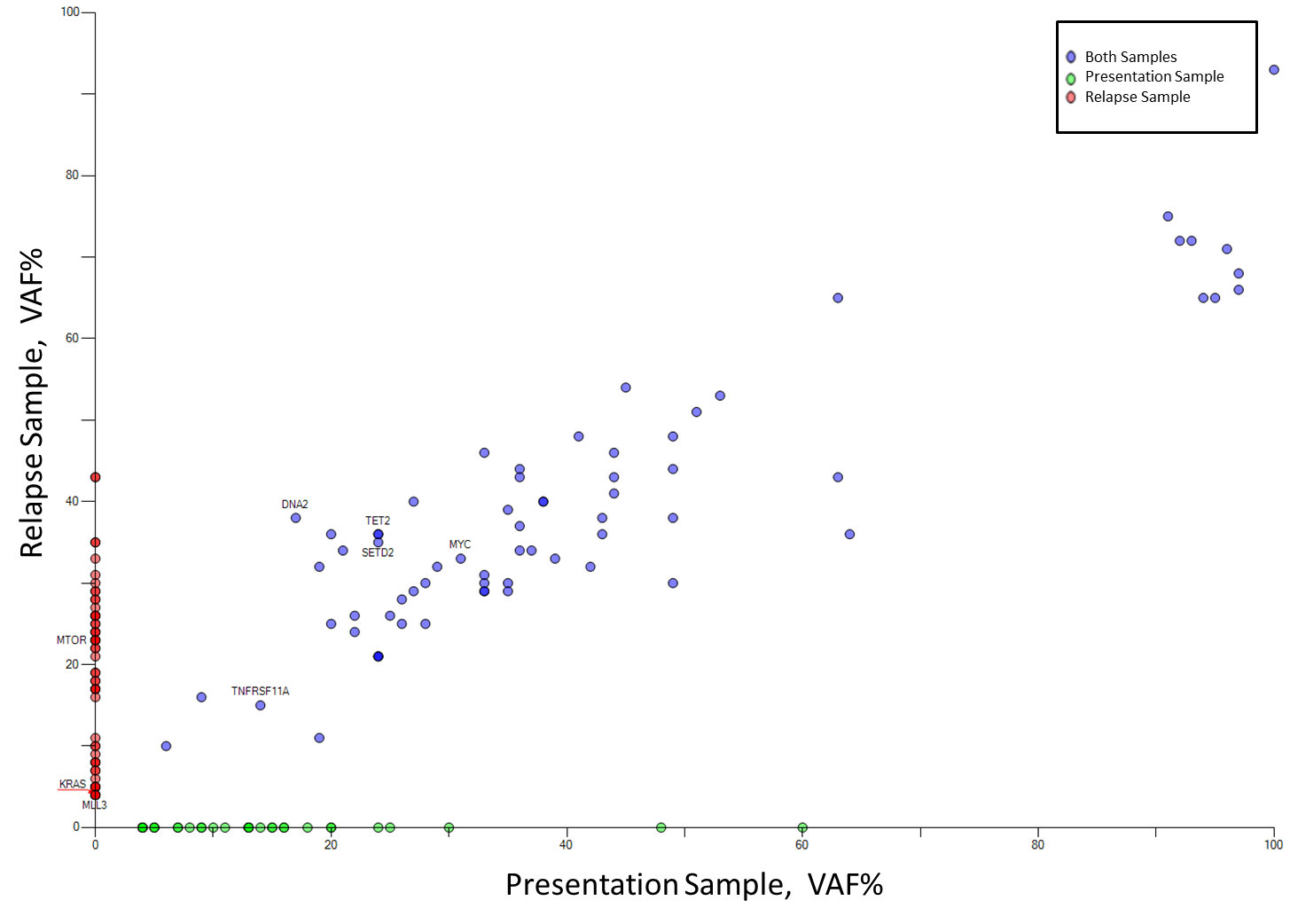

Supplement: Additional File 3 — Scatter plot of paired samples. Displayed are variants in the Presentation on the x-axis compared to Relapse on y-axis. Both the × and y-axes are based on VAF. Variants are colored to indicate whether they are shared or unique. See legend for color assignments. [file 1471-2105-16-S13-S7-S3.png]

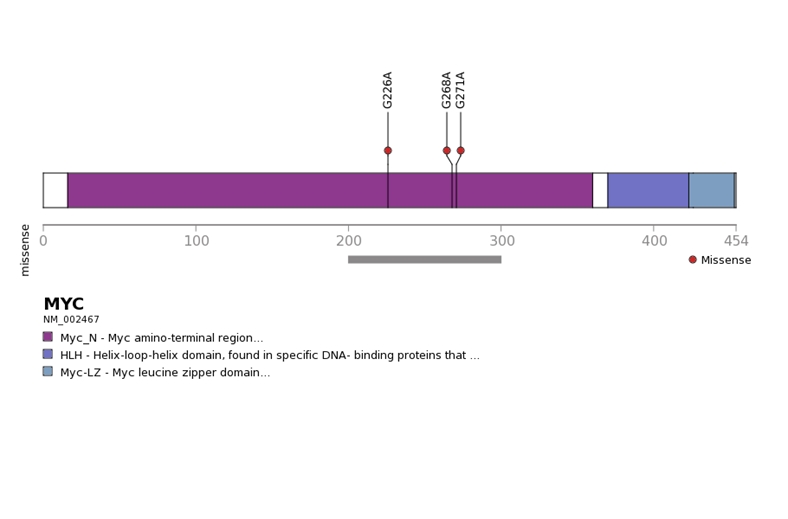

Supplement: Additional File 4 — MYC oncogene with mutation showing possible splicing events. Illustrated is a lolliplot diagram of MYC showing the possible missense mutations in the coding region depending on splicing. [file 1471-2105-16-S13-S7-S4.jpg]

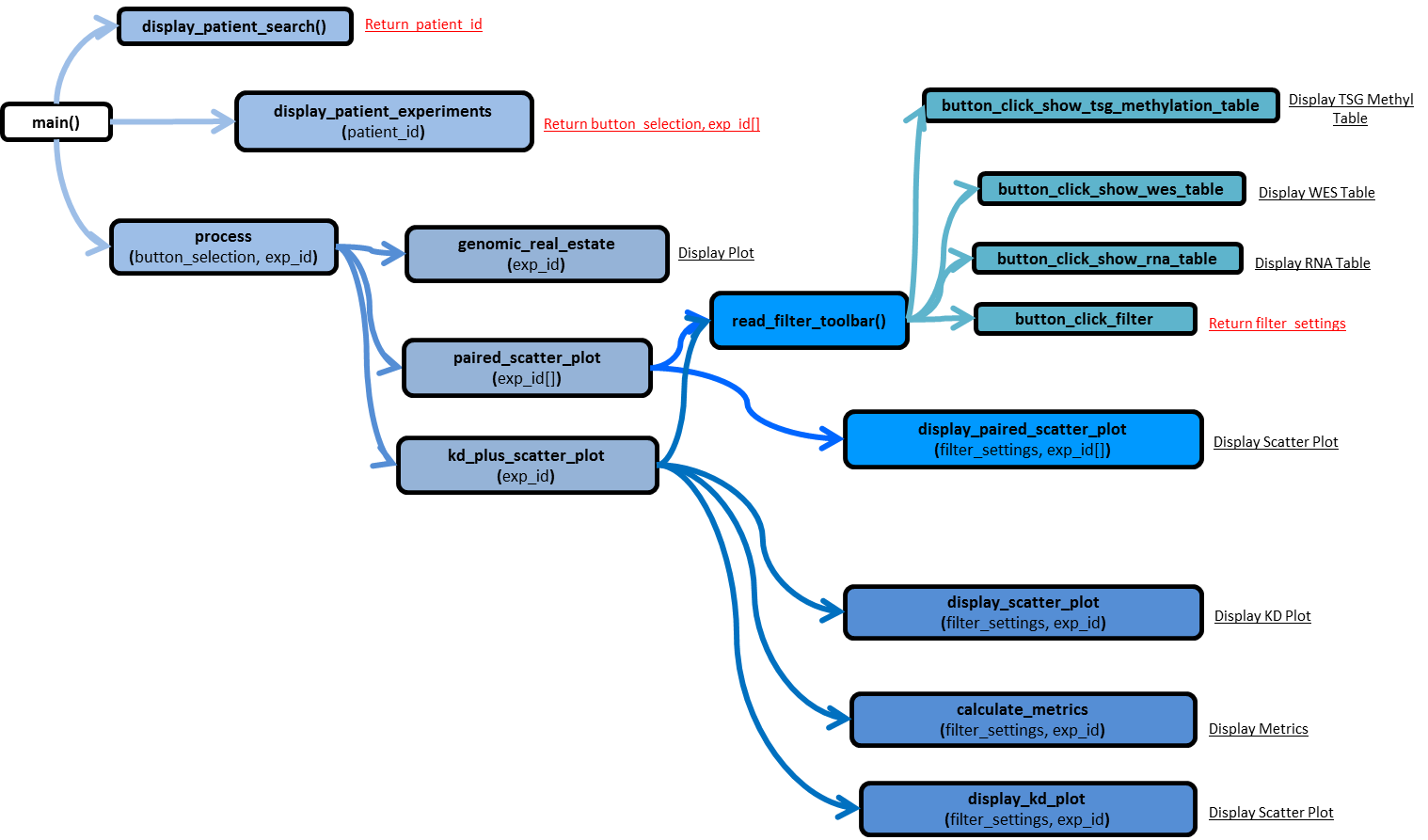

Supplement: Additional File 5 — iCloneViz pseudocode flow diagram. Illustrated is the execution flow of iCloneViz and its associated subroutines. Each subroutine and its formal parameters is represented as a node. Each arc represents a subroutine call from one subroutine to another. [file 1471-2105-16-S13-S7-S5.png]
